# Multiple Targets on the Gln3 Transcription Activator Are Cumulatively Required for Control of Its Cytoplasmic Sequestration

**DOI:** 10.1534/g3.116.027615

**Published:** 2016-03-11

**Authors:** Rajendra Rai, Jennifer J. Tate, Terrance G. Cooper

**Affiliations:** Department of Microbiology, Immunology and Biochemistry, University of Tennessee Health Science Center, Memphis, Tennessee 38163

**Keywords:** Gln3, mTorC1, methionine sulfoximine, nitrogen limitation, rapamycin

## Abstract

A remarkable characteristic of nutritional homeostatic mechanisms is the breadth of metabolite concentrations to which they respond, and the resolution of those responses; adequate but rarely excessive. Two general ways of achieving such exquisite control are known: stoichiometric mechanisms where increasing metabolite concentrations elicit proportionally increasing responses, and the actions of multiple independent metabolic signals that cumulatively generate appropriately measured responses. Intracellular localization of the nitrogen-responsive transcription activator, Gln3, responds to four distinct nitrogen environments: nitrogen limitation or short-term starvation, *i.e.*, nitrogen catabolite repression (NCR), long-term starvation, glutamine starvation, and rapamycin inhibition of mTorC1. We have previously identified unique sites in Gln3 required for rapamycin-responsiveness, and Gln3-mTor1 interaction. Alteration of the latter results in loss of about 50% of cytoplasmic Gln3 sequestration. However, except for the Ure2-binding domain, no evidence exists for a Gln3 site responsible for the remaining cytoplasmic Gln3-Myc^13^ sequestration in nitrogen excess. Here, we identify a serine/threonine-rich (Gln3_477–493_) region required for effective cytoplasmic Gln3-Myc^13^ sequestration in excess nitrogen. Substitutions of alanine but not aspartate for serines in this peptide partially abolish cytoplasmic Gln3 sequestration. Importantly, these alterations have no effect on the responses of Gln3-Myc^13^ to rapamycin, methionine sulfoximine, or limiting nitrogen. However, cytoplasmic Gln3-Myc^13^ sequestration is additively, and almost completely, abolished when mutations in the Gln3-Tor1 interaction site are combined with those in Gln3_477–493_ cytoplasmic sequestration site. These findings clearly demonstrate that multiple individual regulatory pathways cumulatively control cytoplasmic Gln3 sequestration.

A simple rain shower can convert a lush environment inhabited by a *Saccharomyces cerevisiae* yeast cell into a barren landscape, or anything in between, depending on its severity. Coping with such changes requires a rapid response that is adequate but not excessive. Given these demands, it is not surprising that Gln3 and Gat1, the nitrogen-responsive GATA-family transcription activators, are finely regulated and rapidly responsive (Cooper 2004; Broach 2012; Conrad *et al.* 2014). In lush nitrogen-replete conditions, Gln3 and Gat1 are cytoplasmic, and the transcription they mediate highly repressed (Figure 1). Cytoplasmic sequestration correlates with formation of complexes with the negative nitrogen regulator Ure2 (Blinder *et al.* 1996; Bertram *et al.* 2000; Carvalho and Zheng 2003). As nitrogen becomes limiting, or only poorly transported, or catabolized nitrogen sources are to be found, Gln3 and Gat1 move into the nucleus and robustly transcribe genes encoding the catabolic enzymes and transport systems needed to broadly scavenge poor, derepressive nitrogen sources. This overall regulatory phenomenon has long been designated nitrogen catabolite repression (NCR) (Cooper 1982).

**Figure 1:**
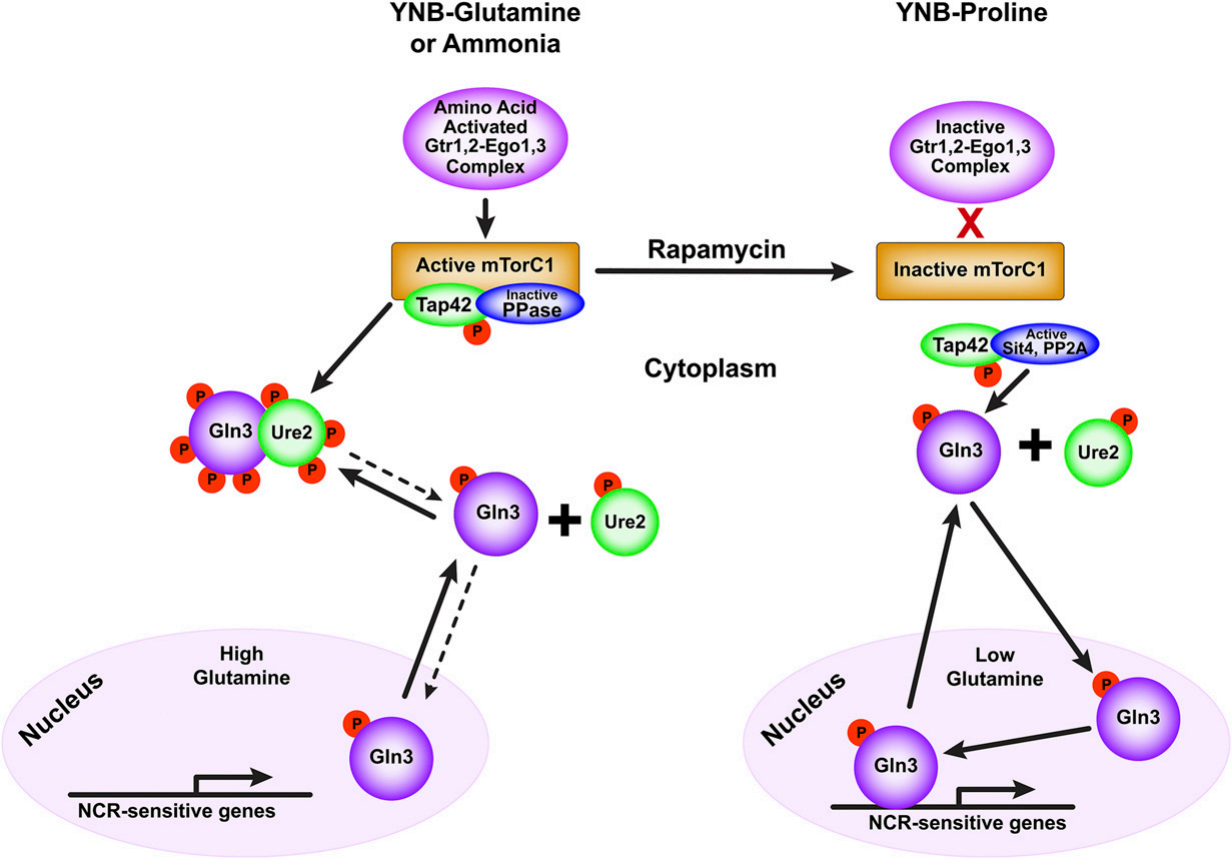
Schematic summarizing nitrogen-responsive regulation of the mTorC1 kinase complex and downstream control of Gln3 localization in nitrogen excess (*left pathway*) or limitation (*right pathway*).

Rapamycin, an inhibitor of the mechanistic target of rapamycin (mTor)—a serine/threonine protein kinase complex (mTorC1)—aberrantly elicits Sit4 phosphatase-dependent Gln3 dephosphorylation, nuclear localization, and Gln3-mediated transcription in nitrogen-rich medium (Figure 1, right yeast pathway) (Beck and Hall 1999; Cardenas *et al.* 1999; Hardwick *et al.* 1999; Bertram *et al.* 2000). These correlations led to the conclusion that NCR-sensitive Gln3 regulation was mediated by mTor activity, Gln3 being cytoplasmic when mTorC1 activity was high, and nuclear when it was low or inhibited by rapamycin.

In nitrogen excess, mTorC1 is activated in an amino acid- and Gtr-Ego complex-dependent manner (Figure 1, left yeast pathway) (Binda *et al.* 2009, 2010; Zhang *et al.* 2012; Jeong *et al.* 2012). Activated mTorC1 phosphorylates Gln3 and Tor associated protein, Tap42, which, in turn, interacts with TorC1, PP2A, and Sit4 phosphatases, (Di Como and Arndt 1996; Jiang and Broach 1999). mTorC1-bound Tap42-Sit4 and Tap42-PP2A complexes are inactive and unable to dephosphorylate Gln3 (Figure 1, left yeast pathway) (Di Como and Arndt 1996; Beck and Hall 1999; Bertram *et al.* 2000; Wang *et al.* 2003; Düval *et al.* 2003; Yan *et al.* 2006). Correlating with these events, Gln3 complexed with Ure2 is sequestered in the cytoplasm. However, even under these conditions, a small amount of Gln3 continues to enter the nucleus, but can exit from it without binding to its target GATA sequences in NCR-sensitive promoters (Rai *et al.* 2015).

Under nitrogen-limiting conditions, the Gtr-Ego complex cannot activate mTorC1 (Rousselet *et al.* 1995; Neklesa and Davis 2009; Spielewoy *et al.* 2010; Wu and Tu 2011; Bonfils *et al.* 2012; Panchaud *et al.* 2013a, 2013b; Laxman *et al.* 2014). These conditions, or rapamycin treatment, permits release and activation of the Tap42-Sit4 complex from mTorC1, and dephosphorylation of Gln3. (Figure 1, right yeast pathway) (Yan *et al.* 2006). Gln3 dissociates from the Gln3-Ure2 complex, cycles into the nucleus and activates transcription (Figure 1, right yeast pathway) (Carvalho and Zheng 2003).

Three observations indicated that NCR-sensitive regulation was more complicated than previously envisioned: (i) Gln3 phosphorylation in nutrient limitation and starvation differed from those generated by rapamycin inhibition of mTorC1 (Cox *et al.* 2004a). (ii) Sit4-dependent Gln3 dephosphorylation was higher in repressive nitrogen-replete medium than in derepressive nitrogen-limiting conditions, the opposite of its predicted behavior (Tate *et al.* 2006). (iii) Rapamycin-elicited, Sit4-dependent, Gln3 dephosphorylation was insufficient for nuclear Gln3 localization in strains lacking PP2A phosphatase (Tate *et al.* 2009, 2010).

While these unexpected observations raised questions about the mechanism of NCR-sensitive Gln3 regulation, it was not until the six independent methods routinely and interchangeably used to downregulate mTorC1 activity were simultaneously analyzed with a single reporter, Gln3-Myc^13^, that the full complexity of overall nitrogen-responsive regulation was appreciated (Tate and Cooper 2013). The six methods were clearly not physiologically equivalent as had been tacitly accepted, because each one exhibited a specific Gln3 response with unique, hierarchal phosphatase requirements (Tate and Cooper 2013). Further, in nitrogen excess, Gln3 relocated to the cytoplasm in the absence of Gtr-Ego complex components required for mTorC1 kinase activation (Figure 1) (Tate *et al.* 2015a). These observations cumulatively demonstrated that Gln3 localization and function were controlled in a highly complex manner involving multiple regulatory pathways.

To deconvolve the complex regulation of Gln3, we needed probes that isolated the individual mechanisms of regulation. To achieve this goal, we reasoned that the existence of multiple regulatory pathways predicted corresponding targets for each of them on the Gln3 molecule itself. Following this reasoning, we localized a Gln3-mTor1 interacting site to 17 Gln3 residues with a predicted propensity to fold into an amphipathic α-helix (Kulkarni *et al.* 2001; Carvalho and Zheng 2003; Rai *et al.* 2013). When this Gln3-mTor1 interaction site was altered, rapamycin no longer elicited nuclear Gln3 localization, but the Gln3 response to poor, or limiting, nitrogen remained fully intact; it was like wild type (Rai *et al.* 2013). However, cytoplasmic Gln3 sequestration was only partially diminished, which indicated that the Gln3-mTor1 interaction site was not acting alone to sequester Gln3 in the cytoplasm. N-terminal of the mTor1 interacting domain, we identified a larger domain specifically required for a Gln3 response to rapamycin-treatment, but not the mTor1-Gln3 interaction (Rai *et al.* 2014). Importantly, aspartate, but not alanine, substitutions throughout the 80-residue domain uniformly abolished rapamycin-responsiveness, but did not affect either cytoplasmic Gln3 sequestration in nitrogen excess, or its response to derepressive nitrogen sources such as proline or allantoin.

Strikingly, none of the above mutant studies identified an effect on Msx-elicited nuclear Gln3 localization. This was disturbing because glutamine was posited to positively control mTor activity, and thereby Gln3 localization and function (Crespo *et al.* 2002). However, dispensibility of Gtr-Ego complex components for maintaining cytoplasmic Gln3 sequestration in nitrogen-rich glutamine medium argued that glutamine probably functioned in some other way downstream of mTorC1 activation. This turned out to be the case. Gln3 cycles into and out of the nucleus (Carvalho and Zheng 2003), and is regulated at both nuclear entry and exit. The overall nitrogen supply of the cell regulates nuclear Gln3 entry, whereas the glutamine level itself, or that of a metabolite that can be mimicked by glutamine analogs, dictates the course of intra-nuclear Gln3 (Rai *et al.* 2015). In high glutamine (growth with glutamine or, to a limited extent, ammonia as sole nitrogen source), any Gln3 that enters the nucleus can cycle out again without binding to its target GATA sequences in NCR-sensitive gene promoters (Figure 1, left nuclear pathway). With any other nitrogen source, including rich YEPD medium, Gln3 also cycles out of the nucleus, but only after binding to its promoter targets (Figure 1, right nuclear pathway) (Rai *et al.* 2015). It is also pertinent that the rare glutamine tRNA_CUG_ is required for nuclear Gln3 localization in nitrogen starved and rapamycin-treated cells (Tate *et al.* 2015b).

In all of the above investigations, an important Gln3 site has been conspicuously missing—one uniquely required for cytoplasmic sequestration in nitrogen excess other than the Ure2 binding site, and one that is independent of responses to rapamycin or Msx. We now report a potential candidate for this site in an unexplored region N-terminal of the sites required for rapamycin-responsiveness and mTor1-binding. It is a highly conserved 15-residue serine/threonine-rich (12 of 15 residues) region flanked by basic residues. Alteration of these serine residues partially abolished cytoplasmic sequestration of Gln3-Myc^13^, but did not affect its responses to rapamycin, Msx treatment, or limiting nitrogen. The fact that the rapamycin- and NCR-responsiveness of Gln3-Myc^13^ remained intact when aspartate or alanine was substituted for these serines argues that they are not the targets of rapamycin-activated Sit4 phosphatase, or limiting nitrogen, and hence represent a novel nitrogen-responsive target in Gln3 that is not part of the mTorC1-Sit4 or Ure2-mediated pathways. Importantly, alteration of these serines, combined with substitutions that destroy the Gln3-mTor1 interaction site, additively and almost completely abolished cytoplasmic Gln3-Myc^13^ sequestration. Thus, we have developed another tool needed to isolate and deconvolve the individual mTorC1-dependent and -independent regulatory pathways that act cumulatively to control cytoplasmic Gln3 sequestration.

## Materials and Methods

### Strains and culture conditions

*Saccharomyces cerevisiae* wild type, JK9-3da (*MATa*, *leu2-3*,*112*, *ura3-52*, *trp1*, *his4*, *rme1*, *HMLa*) or *gln3*Δ, KHC2 (*MATa*, *lys2*, *ura3*, *gln3Δ*::*KanMX*) strains were transformed with wild type and mutant plasmids carrying *gln3* amino acid substitutions. JK9-3da is the parent of TB50, which is the parent of TB123. TCY1 is the parent of KHC2. Unless indicated otherwise, JK9-3da was used as the transformation recipient. Fresh transformants were then analyzed for intracellular Gln3-Myc^13^ distribution. Growth conditions were those described in Tate *et al.* (2009). Cultures (50 ml) were grown to mid-log phase (*A*_600 nm_ = 0.5) in YNB (without amino acids or ammonium sulfate) minimal medium that contained the indicated nitrogen source at a final concentration of 0.1%. Appropriate supplements (120 μg/ml leucine, 20 μg/ml histidine and tryptophan) were added to the medium as necessary to cover auxotrophic requirements. Where indicated, cells were treated with 200 ng/ml rapamycin (+ Rap) for 20 min, or 2 mM methionine sulfoximine (+ Msx) for 30 min. For rapamycin resistance assays, the medium was solid synthetic complete (SC), with or without 50 ng/ml rapamycin.

### Plasmid constructions

*gln3* amino acid substitution mutants were constructed using standard PCR-based methods, and the primer sets in Table 1. The Myc^13^ and *ADH1* transcriptional terminator were derived from pKA62 (Kulkarni *et al.* 2006). The template for all of the constructions, unless otherwise indicated, was pRR536, which contained the wild-type *GLN3* gene, including its native promoter, fused in frame with Myc^13^ at the *GLN3* translational stop codon. All of the constructs were confirmed by DNA sequencing (University of Tennessee Health Science Center Molecular Resource Center DNA sequencing facility).

**Table 1:** Primers used in this work

Plasmid*^a^*	Residue Alterations	Primer Sets
pRR536	Wild Type Gln3_1–730_-Myc^13^	5′-CGCGGATCCTATACCAAATTTTAACCAATCCAATTCGTCAGCAATTGCT-3′
		5′-ATCCCCGCGGGACGTCAACTCCATAGAAGTGACTTTTCCG-3′
pRR676	Gln3_S479A,S480A,S483A,S484A_-Myc^13^_._	5′AACAGATCTGGATGAAGATTTACTGGAACTTGAGGTGTTCGATGAAGTagcagcTCGTCTtgcagcCCTTCTAAAATTAG-3′
		5′-TCTTTATCTAGAGTGATACCTGAAGAAATCATTAGAG-3′
pRR678	Gln3_S479D,S480D,S483D,S484D_-Myc^13^	5′-AACAGATCTGGATGAAGATTTACTGGAACTTGAGGTGTTCGATGAAGTatcatcTCGTCTatcatcCCTTCTAAAATTAG-3′
		5′-TCTTTATCTAGAGTGATACCTGAAGAAATCATTAGAG-3′
pRR680	Gln3_S479A,S480A,S483A,S484A,S486A, S487A,S490A,S491A,S492A,S493A,S495A,S496A, S497A_-Myc^13^	5′-AACAGATCTggctgcagcTTTagcggcagctgcGGTGTTcgctgcAGTagcagcTCGTCTtgcagcCCTTCTAAAATTAG-3
		5′-TCTTTATCTAGAGTGATACCTGAAGAAATCATTAGAG-3′
pRR682	Gln3 _S479D,S480D,S483D,S484D,S486D, S487D,S490D,S491D,S492D,S493D,S495D,S496D, S497D_-Myc^13^	5′-AACAGATCTgtcatcatcTTTatcgtcatcatcGGTGTTgtcatcAGTatcatcTCGTCTatcatcCCTTCTAAAATTAG-3′
		5′-TCTTTATCTAGAGTGATACCTGAAGAAATCATTAGAG-3′
pRR962	Gln3_S469D,T471D,T473D_-Myc^13^	5′-CTAGACTAGTACTACTTCGTCTTGAAGACCTTCTAAAATTAGGgtcAACgtcGTTgtcATTGTGACGCATTAAG-3′
		5′-ATCCCCGCGGGACGTCAACTCCATAGAAGTGACTTTTCCG-3′
		5′-CTAGACTAGTTCGAACACCTCAAGTTCCAGTAAATCTTC-3′
		5′CGCGGATCC TATACCA AATTTTAACC AATCCAATTC GTCAGCAATTGCT-3′
pRR964	Gln3 _S469A,T471A,T473A_ -Myc^13^	5′-CTAGACTAGTACTACTTCGTCTTGAAGACCTTCTAAAATTAGGtgcAACtgcGTTtgcATTGTGACGCATTAAG-3′
		5′-ATCCCCGCGGGACGTCAACTCCATAGAAGTGACTTTTCCG-3
		5′-CTAGACTAGTTCGAACACCTCAAGTTCCAGTAAATCTTC-3′
		5′-CGCGGATCC TATACCA AATTTTAACC AATCCAATTC GTCAGCAATTGCT-3′
pRR1043	Gln3_T485D,S486D,S487D,T489D,S490D, S491D,S492D,S493D_-Myc^13^	5,-GGAACAACAGATCTGGATGAAGATTTtgcggctgctgcggcGTTcgctgctgcACTACTTCGTCTTGAAGACCTTC-3′
		5′-ACATGGTACCATGAGGCCATTATCCTTAAAATCG-3′
pRR1040	Gln3_T485A,S486A,S487A,T489A,S490A, S491A,S492A,S493A_-Myc^13^	5′-GGAACAACAGATCTGGATGAAGATTTatcgtcatcatcgtcGTTgtcatcatcACTACTTCGTCTTGAAGACCTTC-3′
		5′-ACATGGTACCATGAGGCCATTATCCTTAAAATCG-3′
pRR1172	Gln3_S479D,S480D_-Myc^13^	5′-TGGAAGATCTGGATGAAGATTTACTGGAACTTGAGGTGTTCGATGAAGTACTACTTCGTCTgtcgtcCCTTCTAAAATTAG-3′
		5′-TCTTTATCTAGAGTGATACCTGAAGAAATCATTAGAG-3′
pRR1173	Gln3_S496A,S497A_-Myc^13^	5′-CGGAACAACAGATCTggctgcAGATTTACTGGAACTTG-3′
		5′-TCTTTATCTAGAGTGATACCTGAAGAAATCATTAGAG-3′
pRR1178	Gln3_S479A,S480A_-Myc^13^	5′-TGGAAGATCTGGATGAAGATTTACTGGAACTTGAGGTGTTCGATGAAGTACTACTTCGTCTtgcagcCCTTCTAAAATTAG-3′
		5′-TCTTTATCTAGAGTGATACCTGAAGAAATCATTAGAG-3′
pRR1180	Gln3_S496D,S497D_-Myc^13^	5′-CGGAACAACAGATCTgtcgtcAGATTTACTGGAACTTG-3′
		5′-TCTTTATCTAGAGTGATACCTGAAGAAATCATTAGAG-3′
pRR1264	Gln3_L421D,V423D_-Myc^13^	5′-CTAGTCTAGAGTGATACCTGAAGAAATCATTAGAGACAACATCGGTAATACTAATAATATCgacAATgacAATAGGGGAGGC-3′
		5′-CGCGGATCC TATACCA AATTTTAACC AATCCAATTC GTCAGCAATTGCT-3′
pRR1266	Gln3_F430D,S432D,V433D_-Myc^13^	5′-CTAGTCTAGAGTGATACCTGAAGAAATCATTAGAGACAACATCGGTAATACTAATAATATCCTTAATGTAAATAGGGGAGGCTATAACgacAACgacgatCCCTCCCCGGTC-3′
		5′-CGCGGATCC TATACCA AATTTTAACC AATCCAATTC GTCAGCAATTGCT-3′
pRR1268	Gln3_V437D,L438D,M439D_-Myc^13^	5′-CTAGTCTAGAGTGATACCTGAAGAAATCATTAGAGACAACATCGGTAATACTAATAATATCCTTAATGTAAATAGGGGAGGCTATAACTTCAACTCAGTCCCCTCCCCGgacgacgacAACAGCCAATCG-3′
		5′-CGCGGATCC TATACCA AATTTTAACC AATCCAATTC GTCAGCAATTGCT-3′
pRR1270	Gln3_L504E_-Myc^13^	5′-TGGAAGATCTGTTGTTCCGATAgaaCCAAAACCTTCACCTAATAGC-3′
		5′-CGCGGATCC TATACCA AATTTTAACC AATCCAATTC GTCAGCAATTGCT-3′
pRR1272	Gln3_K506E_-Myc^13^	5′-TGGAAGATCTGTTGTTCCGATATTACCAgaaCCTTCACCTAATAGC-3′
		5′-CGCGGATCC TATACCA AATTTTAACC AATCCAATTC GTCAGCAATTGCT-3′
pRR1331	Gln3_N450D,F451D_-Myc^13^	5′-CTGGACTAGTACTACTTCGTCTTGAAGACCTTCTAAAATTAGGAGTAACAGTGTTCGAATTGTGACGCATTAAGTTATTAGAATTCAAATTTGCATTGCTTGCTCCATTgtcatcTGCGTTACTAC-3′
		5′-ATCCCCGCGGGACGTCAACTCCATAGAAGTGACTTTTCCG-3′
		5′-TAGTACTAGTTCGAACACCTCAAGTTCCAGTAAATC-3′
		5′-CGCGGATCC TATACCA AATTTTAACC AATCCAATTC GTCAGCAATTGCT-3′
pRR1323	Gln3_L459D,L464D,M465D_-Myc^13^	5′-CTGGACTAGTACTACTTCGTCTTGAAGACCTTCTAAAATTAGGAGTAACAGTGTTCGAATTGTGACGgtcgtcGTTATTAGAATTgtcATTTGCATTGCTTG-3′
		5′-ATCCCCGCGGGACGTCAACTCCATAGAAGTGACTTTTCCG-3′
		5′-TAGTACTAGTTCGAACACCTCAAGTTCCAGTAAATC-3′
		5′-CGCGGATCC TATACCA AATTTTAACC AATCCAATTC GTCAGCAATTGCT-3′

a All plasmids contain full-length *gln3* genes driven by the native GLN3 promoter.

### Indirect immunofluorescence microscopy and intracellular Gln3-Myc^13^ distribution

Cell collection and immunofluorescent staining were performed as previously described (Cox *et al.* 2004a, 2004b; Tate *et al.* 2006; Georis *et al.* 2008; Tate *et al.* 2009). Cells were imaged with a Zeiss Axioplan 2 imaging microscope, 63 × and 100 × Plan-Apochromat 1.40 oil objectives at room temperature. Images were captured by a Zeiss Axio camera, and AxioVision 3.0 and 4.8.1 (Zeiss) software. As reasoned in Rai *et al.* (2013), it is not possible to objectively determine the intracellular distribution of a protein from images of subjectively chosen fields containing a few cells unless the protein is confined exclusively to a single cellular compartment, *i.e.*, cytoplasmic only or nuclear only. Gln3 is often simultaneously located in both cellular compartments. Therefore, we quantified intracellular Gln3 distribution by manually scoring Gln3-Myc^13^ localization in 200 or more cells in multiple, randomly chosen fields. Scoring was performed exclusively using unaltered, primary .zvi image files generated by the camera, and viewed with Zeiss AxioVision 3.0 and 4.8.1 software. Cells were classified into one of three categories: cytoplasmic (cytoplasmic Gln3-Myc^13^ fluorescence only; red bars), nuclear-cytoplasmic (Gln3-Myc^13^ fluorescence appearing in the cytoplasm as well as colocalizing with DAPI-positive material, DNA; yellow bars), and nuclear (Gln3-Myc^13^ fluorescence colocalizing only with DAPI-positive material; green bars). “Standard” images, that depict the characteristics of these categories, appear in Figure 6 in Tate *et al.* 2009. All conclusions in this work were based on these quantitative data.

**Figure 2:**
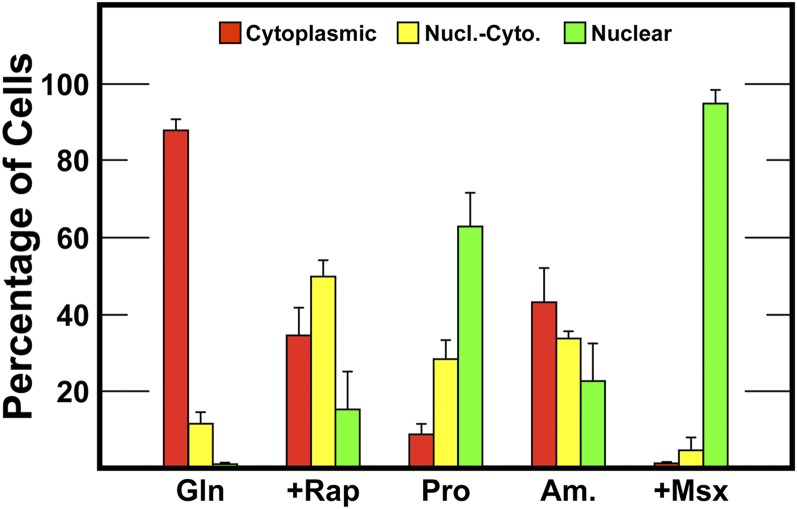
Reproducibility of the data in this work. The histograms represent the means of the eight localization experiments contained in this work. They were performed over 4 yr, measuring Gln3_1-730_-Myc^13^ localization in wild-type JK9-3da transformed with pRR536; error bars represent SD around these means.

The precision of our scoring has been repeatedly documented (Tate *et al.* 2006, 2010; Tate and Cooper 2007). Since wild-type controls accompanied all of the experiments presented below, these data were used to assess the reproducibility of our assays under all of the growth conditions analyzed. Data depicted in Figure 2 represent the means of eight experiments performed over 4 yr, measuring Gln3_1-730_-Myc^13^ localization in wild-type JK9-3da transformed with pRR536; error bars represent the SD around these means.

Images accompanying the histograms in subsequent figures were chosen on the basis that they exhibited intracellular Gln3-Myc^13^ distributions as close as possible to those observed in the quantitative scoring, and are for illustrative purposes only. The images (after conversion from .zvi to .tif files) were processed in the following way using Adobe Photoshop and Illustrator programs: (i) Any alteration of an image was uniformly applied to the entire image presented. (ii) The only alteration of the images was to decrease the background fluorescence due to leak-through light not removed by the barrier filter of the microscope. This avoided any change or loss in cellular detail relative to what was observed in the microscope. The alteration [level settings (shadow and highlight only), gamma settings were never altered] was applied to each image individually. The alterations were nearly the same from one image to another, but were not rigorously identical because the amount of leak-through fluorescence was not identical from one image to another.

### Western blots

Western blots were performed as described earlier (Tate *et al.* 2009; Rai *et al.* 2013).

### Data availability

Following publication, we will expeditiously share all cited strains and plasmids emanating from work supported by this grant with other investigators (for noncommercial use only) upon request. This will be done in accordance with NIH guidelines.

## Results

### Gln3-Myc^13^ phosphorylation profiles of truncation mutants

**Figure 3:**
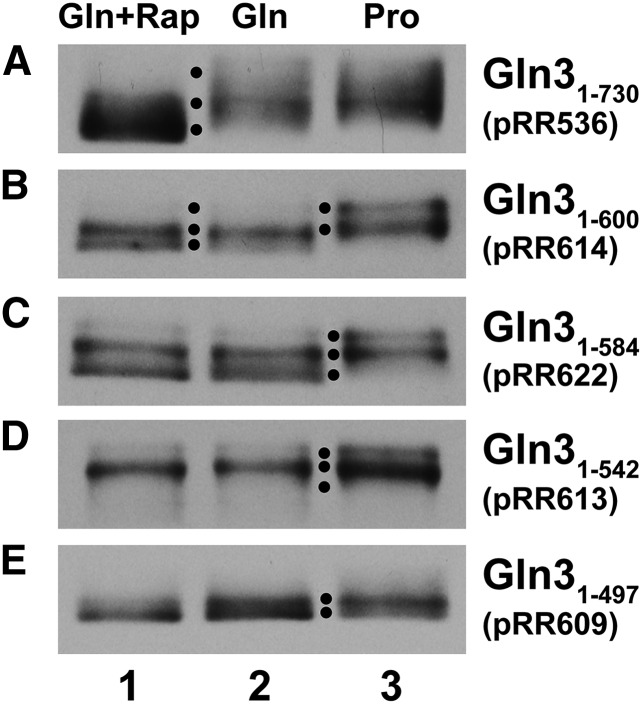
Gln3-Myc^13^ phosphorylation profiles in transformants containing wild type (pRR536) and C-terminal *gln3* truncation mutants. Cells were cultured in untreated YNB-glutamine (Gln) or proline (Pro) media; rapamycin (+Rap) was added where indicated. Western blots were performed as described in *Materials and Methods*. The length of the Gln3 proteins assayed appear above the plasmid numbers. Black dots indicate positions of major species. (A) pRR536, (B) pRR614, (C) pRR622, (D) pRR613, and (E) pRR609.

Previously reported data predict the existence of a unique, rapamycin-independent, Gln3 site required for its response to the cell’s overall nitrogen supply, *i.e.*, NCR. To search for this site, we compared the gross phosphorylation profiles of large Gln3-Myc^13^ truncations in rapamycin-treated cells, and those provided with a repressive (glutamine) *vs.* a derepressive (proline) nitrogen source (Figure 3) (Rai *et al.* 2013, 2014). Wild-type Gln3_1–730_-Myc^13^, and a Gln3_1–600_-Myc^13^ truncation, yielded three species in western blots (see black dots in each panel): the amount of the most rapidly migrating Gln3 species increased at the expense of the middle species; a normal response to rapamycin (Figure 3, A and B). A slower migrating upper species appeared only in proline-grown cells (Figure 3B). Three species were also present in a Gln3_1–584_-Myc^13^ truncation, but the most rapidly migrating species no longer changed in intensity when rapamycin was added (Figure 3C). With a Gln3_1–542_-Myc^13^ truncation, the proline-responsive and middle species were both still present (Figure 3D). However, the third species corresponding to that obtained with rapamycin-treatment was abolished, thus supporting the conclusions of our earlier investigations that this region was required for a response to rapamycin (Rai *et al.* 2014). Finally, in a Gln3_1–497_-Myc^13^ truncation, two bands were present, neither of which was proline-responsive (Figure 3E). The loss of the proline-response occurred at a position just N-terminal to residues previously shown to be specifically required for a response to rapamycin, 510–594 (Rai *et al*. 2014).

### C-Terminal region of Gln3 is predicted to be highly disordered

The above observations raised the possibility that a Gln3 region required for its NCR-sensitive response might be situated N-terminal of Gln3 residue 510. Given that the two Gln3 regions previously shown to be required for stimulus-specific nuclear Gln3 localization were associated with residues exhibiting predicted propensities to fold into α-helices, we analyzed the secondary structure of this region with multiple programs including PrDos, and Protein DisOrder Prediction (http://prdos.hgc.jp/cgi-bin/top.cgi; Ishida and Kinoshita 2007).

**Figure 4:**
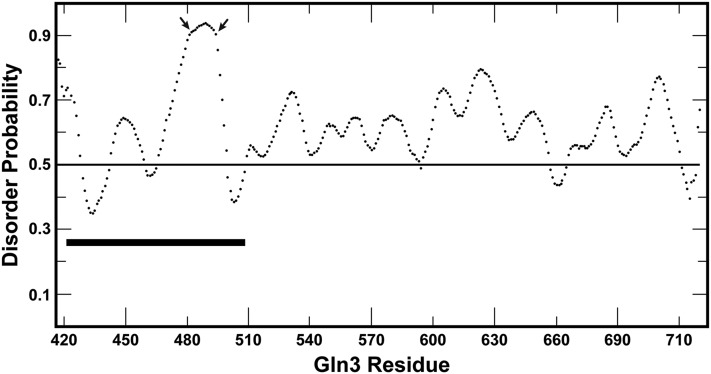
Plot of the predicted probability of Gln3_420–730_ being disordered. The cutoff for predictions exhibiting a 5% or less probability of generating a false positive is indicated (line at 0.5). The bold black line indicates the region of Gln3 analyzed in this work.

**Figure 5:**
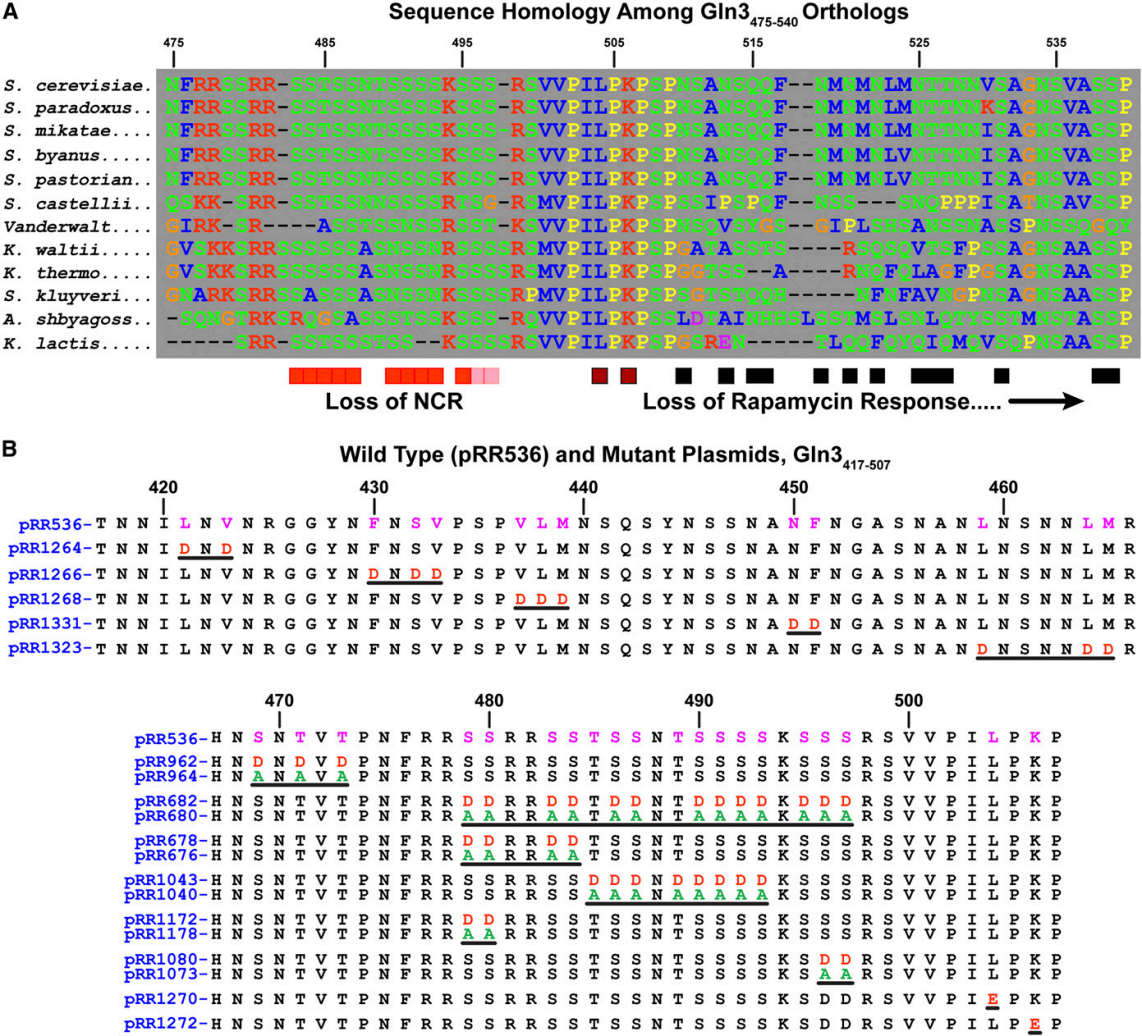
(A) Region of Gln3 (residues 475–540) that exhibits high homology among 11 strains most related to *Saccharomyces cerevisiae*. The residue designations are color-coded: red, basic; blue, hydrophobic; green, polar; orange, glycine; yellow, proline and purple, acidic. Boxes beneath the homology series indicate residues shown to be required for cytoplasmic Gln3-Myc^13^ sequestration, red; a response to rapamycin, black. Substitutions that affected both of these responses are indicated in red-black, and those with very modest effects in light red/pink. (B) Gln3 amino acid substitutions analyzed in this work. Plasmid numbers appear to the left of the sequences. All residues substituted are indicated in pink in the wild type sequence (pRR536) that appears at the top of each panel. Black lines highlight the regions where substitutions were made.

The results were dramatic. Most of the C-terminal half of Gln3 exhibited a predicted probability that ranged from 50% to greater than 90% of being disordered (prediction false positive rate ∼ 5%) (Figure 4). Further, this region of Gln3 contained a 65-residue region highly conserved among the 11 yeast species most related to *S. cerevisiae* (Figure 5A). A portion of this conserved region is rich in both basic amino acids and serine/threonine residues, characteristics of some protein kinase sites (Figure 5A). Given these observations, and the fact that nothing was known about the functional significance of Gln3 residues in this region, we prepared a series of full length Gln3_1-730_-Myc^13^ *CEN*-based constructs (driven by the native Gln3 promoter), in which aspartate or alanine was substituted for various residues (Figure 5B). The ability of this assay system to faithfully reproduce Gln3 regulation has been repeatedly documented (Rai *et al.* 2014, 2015).

### Alteration of a region rich in hydrophobic, asparagine and glutamine residues has no effect on stimulus-specific Gln3 responses

**Figure 6:**
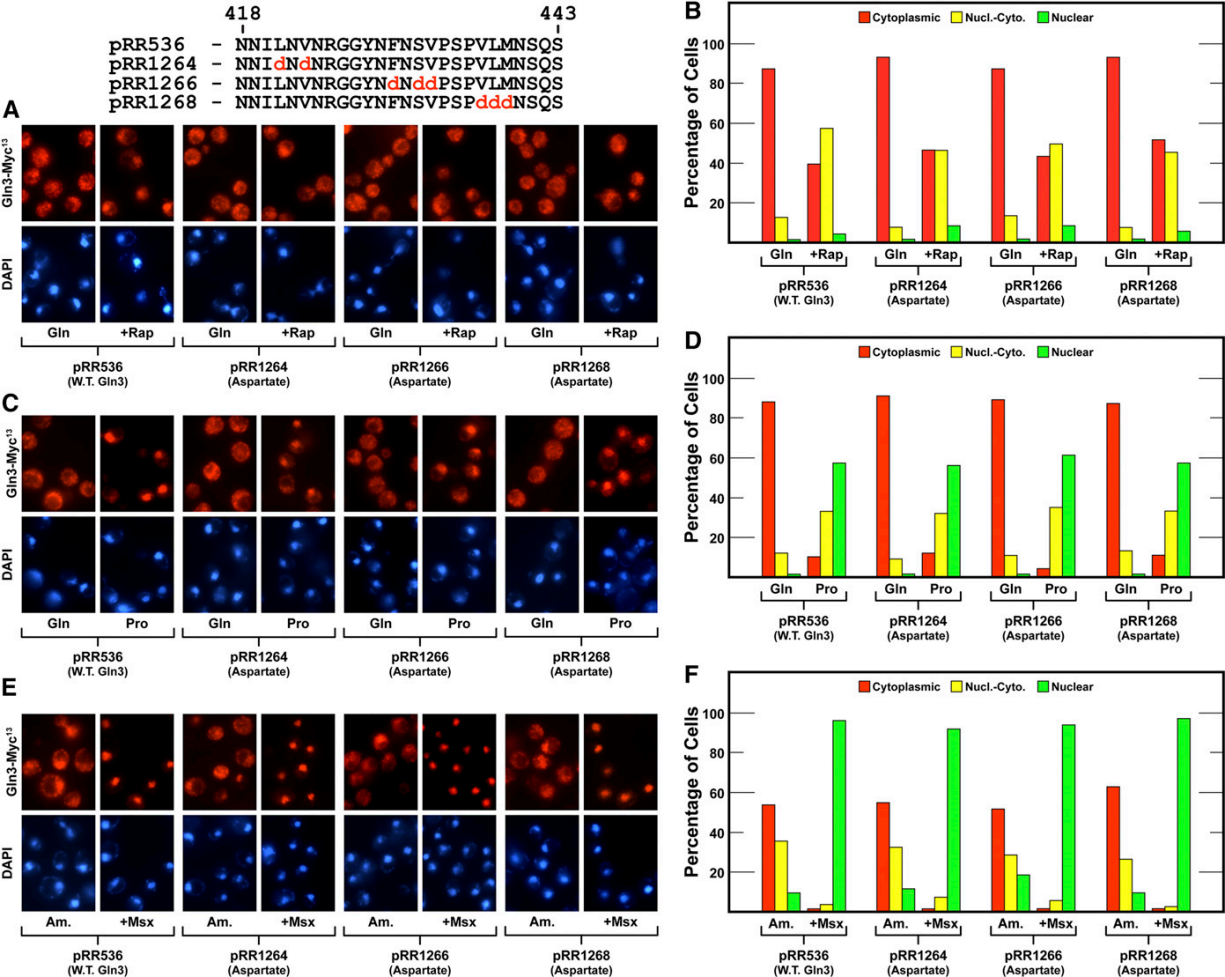
The response of Gln3-Myc^13^ localization to aspartate substitutions for hydrophobic amino acids in Gln3 region 421 to 439. (A) and (B) Cells were cultured in YNB-glutamine medium (Gln); rapamycin (200 ng/ml) was added where indicated (+ Rap) for 20 min. (C) and (D) Cells were cultured in either YNB-glutamine or proline medium. (E) and (F) Cells were cultured in YNB-ammonia (Am.); Msx (2 mM final concentration) was added for 30 min where indicated (+ Msx). Intracellular Gln3-Myc^13^ localization was scored as indicated in *Materials and Methods* as being cytoplasmic (red bars), nuclear-cytoplasmic (yellow bars), or nuclear (green bars). The mutant amino acid substitutions are shown above (A) and in context in Figure 5B.

**Figure 7:**
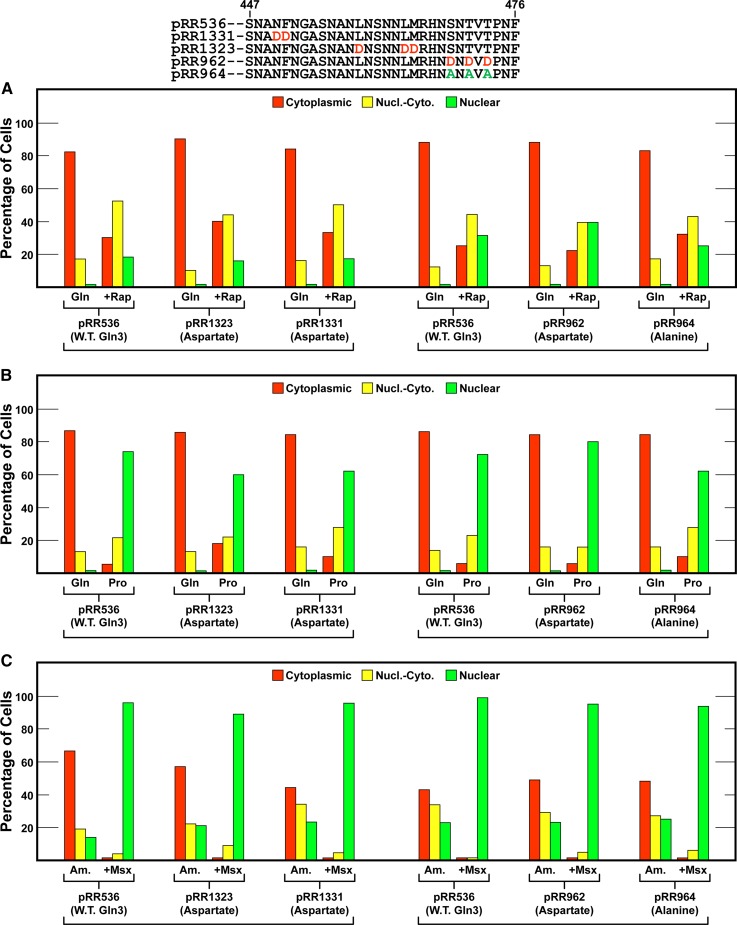
(A–C) show the response of Gln3-Myc^13^ localization to substitution of amino acids in Gln3 region 450 to 473. The format of the experiments and presentation of the data were as in Figure 6.

The first region analyzed was between Gln3 residues 421 and 439. It contained 28% hydrophobic, and 32% asparagine/glutamine residues. We replaced two or three hydrophobic residues in each of three constructs with charged aspartate residues [Gln3_L421D,V423D_-Myc^13^ (pRR1264); Gln3_F430D,S432D,V433D_-Myc^13^ (pRR1266); Gln3_V437D,L438D,M439D_-Myc^13^ (pRR1268)], reasoning that, if this region interacted with other proteins, or its secondary structure was needed for a particular function, these alterations would damage it. In all three substitution mutants, Gln3-Myc^13^ localization was indistinguishable from wild type, irrespective of whether we assessed glutamine- *vs.* proline-grown cells, or cells treated with rapamycin or Msx (Figure 6, C and D, A and B, and E and F, respectively). The same result was observed when we substituted aspartate for the hydrophobic residues between Gln3 residues 450 and 465 [Gln3_N450D,F451D_-Myc^13^ (pRR1331); Gln3_L459D,L464D,M465D_-Myc^13^ (pRR1323)] (Figure 7, left half).

### Gln3 region, predicted to be disordered, is required for cytoplasmic Gln3 sequestration under repressive growth conditions

We then shifted our attention to Gln3_469–499_, a region predicted to be disordered (Figure 4, > 90%) and exhibiting conservation among related species. The first pair of mutants constructed were Gln3_S469D,T471D,T473D_-Myc^13^ (pRR962) and Gln3_S469A,T471A,T473A_-Myc^13^ (pRR964) (Figure 5B). These choices derived from an earlier phosphoproteomics report that these residues were phosphorylated (Swaney *et al.* 2013). The aspartate and alanine substitutions, however, had no effect on Gln3-Myc^13^ localization, irrespective of the growth conditions employed (Figure 7, right half). In summary, the above constructs indicated that integrity of the region between Gln3 residues 421 and 473 was not demonstrably necessary for wild type regulation of Gln3 localization.

**Figure 8:**
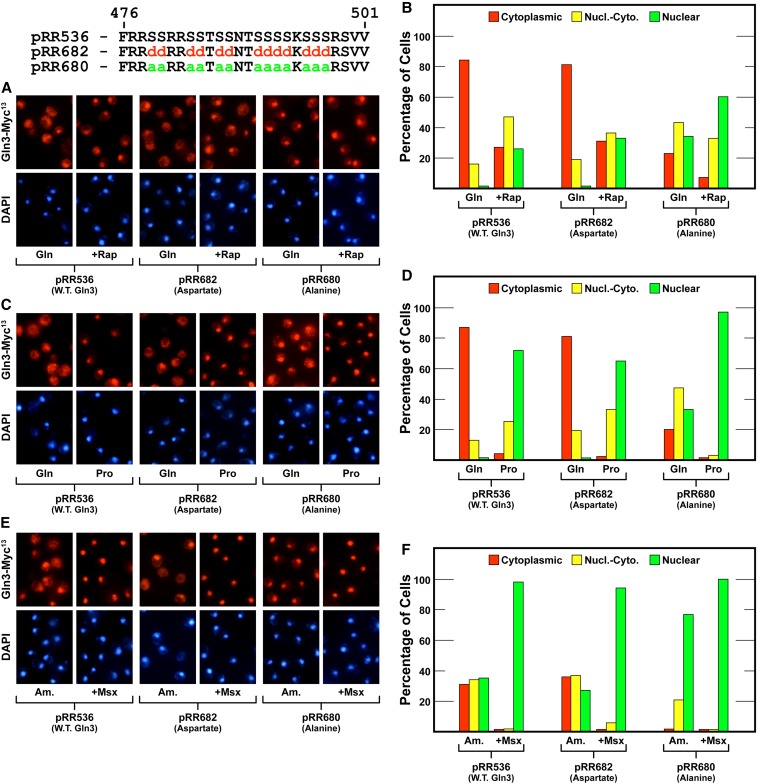
Substitution of alanine for 13 serine residues in the serine/threonine/basic amino acid-rich region of Gln3 largely abolishes cytoplasmic sequestration of Gln3-Myc^13^ (pRR680) in nitrogen-rich glutamine (A–D) or ammonia (E and F) medium. Aspartate substitutions (pRR682) elicited Gln3-Myc^13^ intracellular Gln3-Myc^13^ distributions that were indistinguishable from wild type. The experimental format and presentation of the data were as described in Figure 6.

The next construct, however, was much more informative. We substituted aspartate (pRR682) or alanine (pRR680) for 13 serine/threonine residues situated between Gln3 positions 477 and 497 (Figure 8). These serines are embedded in the conserved basic/serine-rich portion of this region (Figure 5A). Aspartate substitutions had little to no demonstrable effect on Gln3-Myc^13^ localization. It was virtually wild type in all conditions assayed (Figure 8, pRR682). In contrast, substituting alanine for these serines strikingly diminished the ability of the cell to sequester Gln3-Myc^13^ in the cytoplasm of glutamine-grown cells. In fact, the intracellular distribution of Gln3-Myc^13^ in a glutamine-grown pRR680 transformant was almost the same as a similarly grown wild-type transformant treated with rapamycin, or one in which the Gln3-mTor1 interaction site was abolished (Figure 8B, compare W.T. Gln + Rap with pRR680 Gln) (Rai *et al.* 2013). This diminished ability for cytoplasmic sequestration was reflected in correspondingly greater nuclear Gln3-Myc^13^ localization in rapamycin-treated cells (Figure 8, A and B, pRR680) and those grown in derepressive proline media (Figure 8, C and D, pRR680). The greatest effect, however, was observed in ammonia-grown cells, in which Gln3-Myc^13^ was nearly undetectable in the cytoplasm, or the nuclear-cytoplasmic scoring category, in a substantial majority of the cells (Figure 8, E and F).

Three characteristics of the pRR680 substitution mutant merit emphasis: (i) it retained its ability to respond to rapamycin, arguing that these residues were not required for a response to rapamycin inhibition of TorC1 kinase. (ii) As extensive as the alanine substitutions were in this region of Gln3, they failed to totally abolish the ability of Gln3-Myc^13^ to be retained in the cytoplasm when cells were grown in repressive medium. (iii) Neither aspartate nor alanine substitutions in this region had any effect on nuclear Gln3-Myc^13^ localization in Msx-treated cells, just as has occurred in all of the Gln3 C-terminal alterations previously reported (Figure 8, E and F) (Rai *et al*. 2013, 2014). In pRR680, however, the fold-effect of Msx addition was small because Gln3-Myc^13^ was already mostly nuclear.

**Figure 9:**
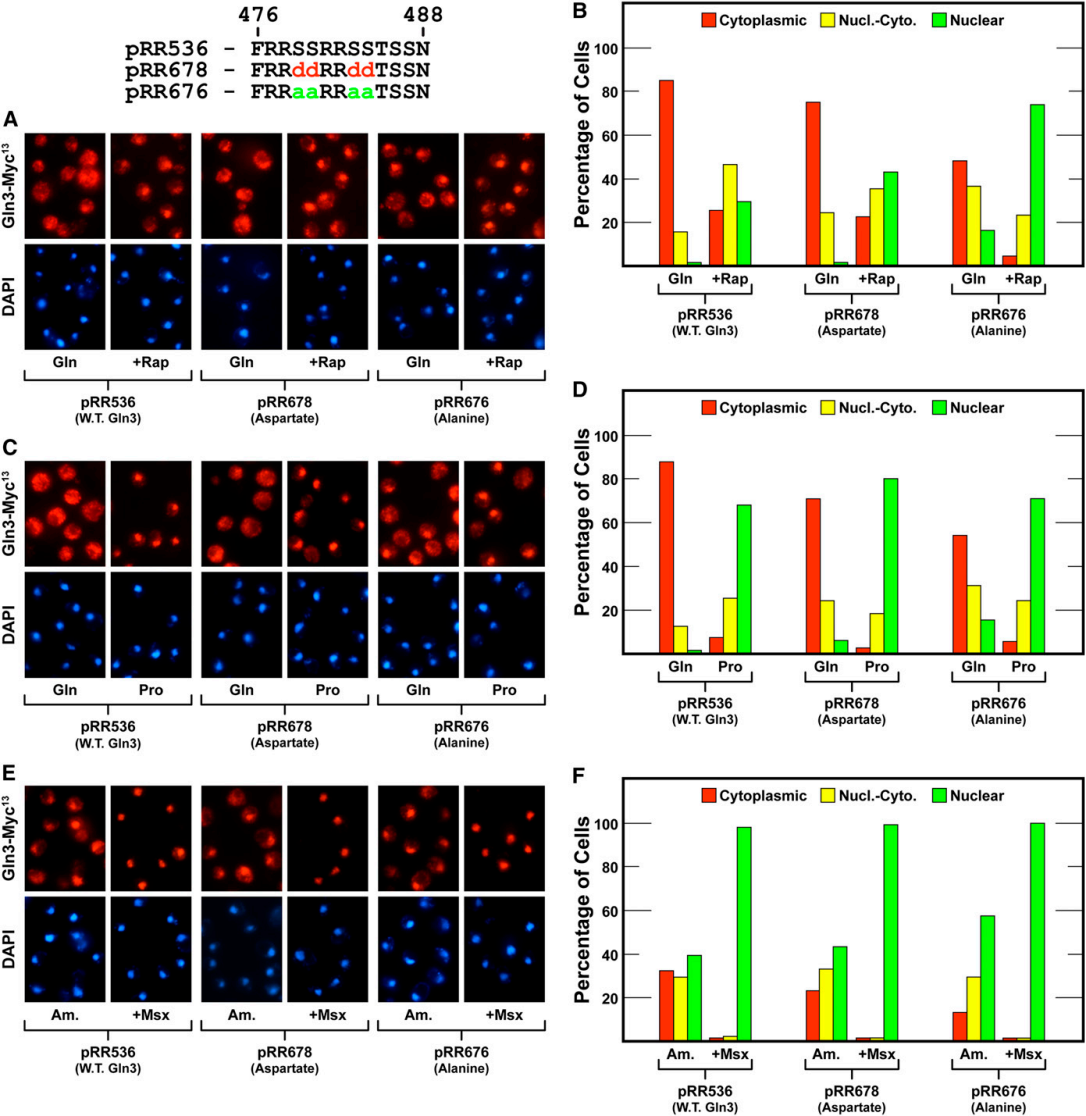
Substitution of alanine for the N-terminal four serine residues in the serine/threonine/basic amino acid-rich region of Gln3 reduces cytoplasmic sequestration of Gln3-Myc^13^ (pRR676) in nitrogen-rich glutamine medium (A–D), and largely abolishes it in ammonia medium (E and F). Aspartate substitutions (pRR678) elicited Gln3-Myc^13^ intracellular Gln3-Myc^13^ distributions that were indistinguishable from wild type. The experimental format and presentation of the data were as described in Figure 6.

The strength of the serine-to-alanine substitution phenotype prompted us to further localize the residues upon which this effect depended. To this end, we substituted aspartate or alanine for four serine residues contained in an RRSSRRSS repeat (Gln3_S479D,S480D,S483D,S484D_-Myc^13^, pRR678; Gln3_S479A,S480A,S483A,S484A_-Myc^13^, pRR676) (Figure 9). Here, as with pRR680, the aspartate substitutions yielded a wild type phenotype (Figure 9, pRR678). In contrast, alanine residues yielded a similar, but somewhat muted, version of the phenotype observed with the 13 substitution construct (Figure 9, pRR676). Cytoplasmic Gln3-Myc^13^ sequestration decreased by about half in glutamine-grown cells when compared to wild type (Figure 9, A and B, and C and D). Although nuclear Gln3-Myc^13^ localization clearly increased in ammonia-grown cells, the shift was also not as dramatic as with pRR680.

**Figure 10:**
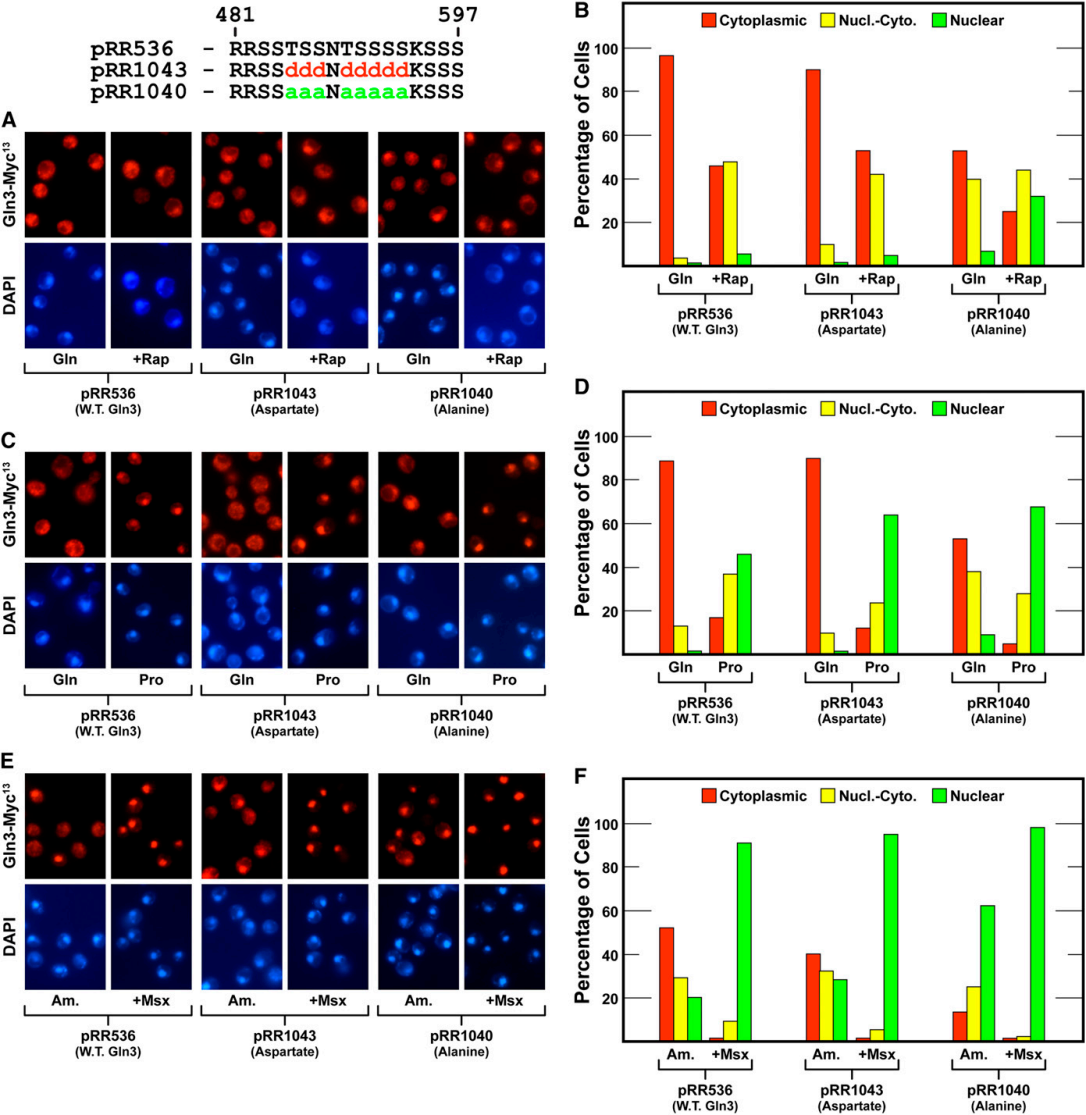
Substitution of alanines for the center eight serine residues in the serine/threonine/basic amino acid-rich region of Gln3 reduces cytoplasmic sequestration of Gln3-Myc^13^ (pRR1040) in nitrogen-rich glutamine medium (A–D) and largely abolishes it in ammonia medium (E and F). Aspartate substitutions (pRR1043) elicited Gln3-Myc^13^ intracellular Gln3-Myc^13^ distributions that were indistinguishable from wild type. The experimental format and presentation of the data were as described in Figure 6.

We next substituted the center eight serines/threonines between Gln3 residues 485 and 493 (pRR1040 and pRR1043). These residues were not flanked by basic amino acids (Figure 5B). Alteration of these residues yielded results similar to those just described for plasmids pRR678 and pRR676 (Figure 10). So they too contributed to the overall diminishment of cytoplasmic Gln3-Myc^13^ sequestration, but again were insufficient to achieve the robust phenotype exhibited by the pRR680 mutant. Therefore, while alteration of serine/threonine residues throughout this region significantly contributed to the loss of cytoplasmic Gln3-Myc^13^ sequestration, it appeared that most or all had to be altered to achieve a maximum effect.

### Alanine substitutions in serine-rich region alter the Gln3-Myc^13^ phosphorylation profile

**Figure 11:**
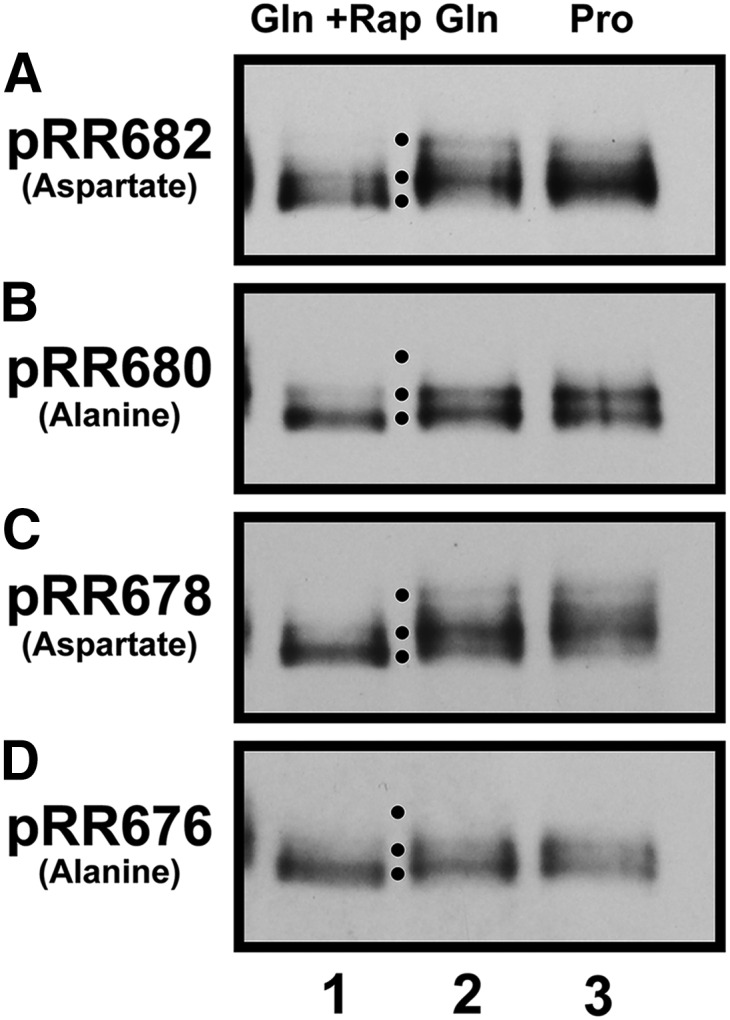
Effects of serine to aspartate [pRR682 (A) and pRR678 (C)], or alanine [pRR680 (B) and pRR676 (D)], subsitutions on Gln3-Myc^13^ electrophoretic mobilities in glutamine (Gln)- or proline (Pro)-grown cells, and glutamine-grown cells treated with rapamycin. Black dots indicate positions of the major species.

The observation that serine to alanine substitutions in the serine-rich region of Gln3 (pRR680) largely abolished cytoplasmic Gln3-Myc^13^ localization prompted us to query whether this result might be associated with an altered ability to phosphorylate Gln3. We particularly wanted to know whether these alterations affected rapamycin-elicited Gln3 dephosphorylation. To this end, we grew four of the above mutants (pRR682, pRR680, pRR678, and pRR676) in proline or glutamine medium, and further treated glutamine-grown cells with rapamycin (Beck and Hall 1999; Cox *et al.* 2004a). In all four cases, the most rapidly migrating Gln3-Myc^13^ species increased in rapamycin-treated cells (Figure 11, A–D, lanes 1, 2, lowest black dot). This indicated that rapamycin-elicited Gln3-Myc^13^ dephosphorylation was unaffected by either the aspartate or alanine substitutions, supporting the conclusion reached from the Gln3-Myc^13^ localization data. In contrast, the Gln3-Myc^13^ phosphorylation profiles observed with serine to aspartate substitutions (pRR682 and pRR678) differed from those obtained with the alanine substitutions (pRR680 and pRR676) (Beck and Hall 1999; Cox *et al.* 2004a). Slower migrating Gln3-Myc^13^ species were observed in untreated cells containing the aspartate substitutions, but were missing in those containing the alanine substitutions (Figure 11, uppermost black dot).

### Alterations of sequences homologous to protein kinase target sites are without effect

**Figure 12:**
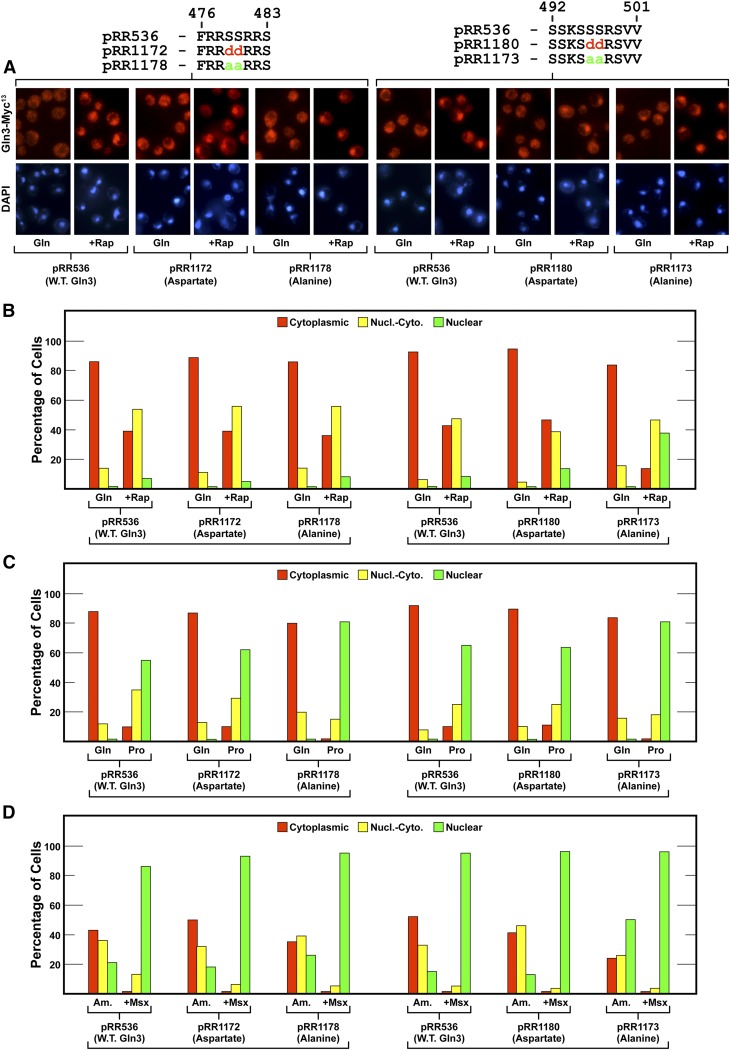
(A–D) Alanine (pRR1178 and pRR1173) or aspartate (pRR1172 and pRR1180) substitutions for serine residues in the serine/threonine/basic amino acid-rich region of Gln3 with homology to known protein kinase phosphorylation sites. The experimental format and presentation of the data were as described in Figure 6.

Two short sequences containing basic and serine residues observed in some protein kinase target sites were situated within the region covered by the 13 substitutions in plasmids pRR682 and pRR680. To specifically determine whether these sites contribute to the regulation of Gln3 localization, we substituted aspartate or alanine for the serines contained in them. The first two serines analyzed were Gln3_S479D,S480D_-Myc^13^, pRR1172 and Gln3_S479A,S480A_-Myc^13^, pRR1178). Gln3-Myc^13^ localization in these substitution mutants was indistinguishable from wild type (Figure 12, left side). We then created aspartate and alanine substitutions at Gln3_S496D,S497D_-Myc^13^ (pRR1080) and Gln3_S496A,S497A_-Myc^13^ (pRR1173). The only convincing effect the alanine substitutions elicited was in ammonia-grown cells (Figure 12). This argued that they participated in cytoplasmic Gln3 sequestration, but in only a minor way.

### Serine to alanine substitutions in the Gln3 RRSSRRSS sequence generate rapamycin hyper-sensitivity

The above data generated a testable prediction. If serines in the Gln3_477–497_ region were truly required for cytoplasmic sequestration, then replacing them with alanine should increase the rapamycin sensitivity of the mutant transformants. Recall that cytoplasmic Gln3 sequestration is lost in *ure2*Δ mutants, where Gln3 is constitutively nuclear and the mutants are hypersensitive to rapamycin (Bertram *et al.* 2000). In contrast, *gln3*Δ mutants are rapamycin resistant. Therefore, we assessed the rapamycin sensitivity of our transformants.

**Figure 13:**
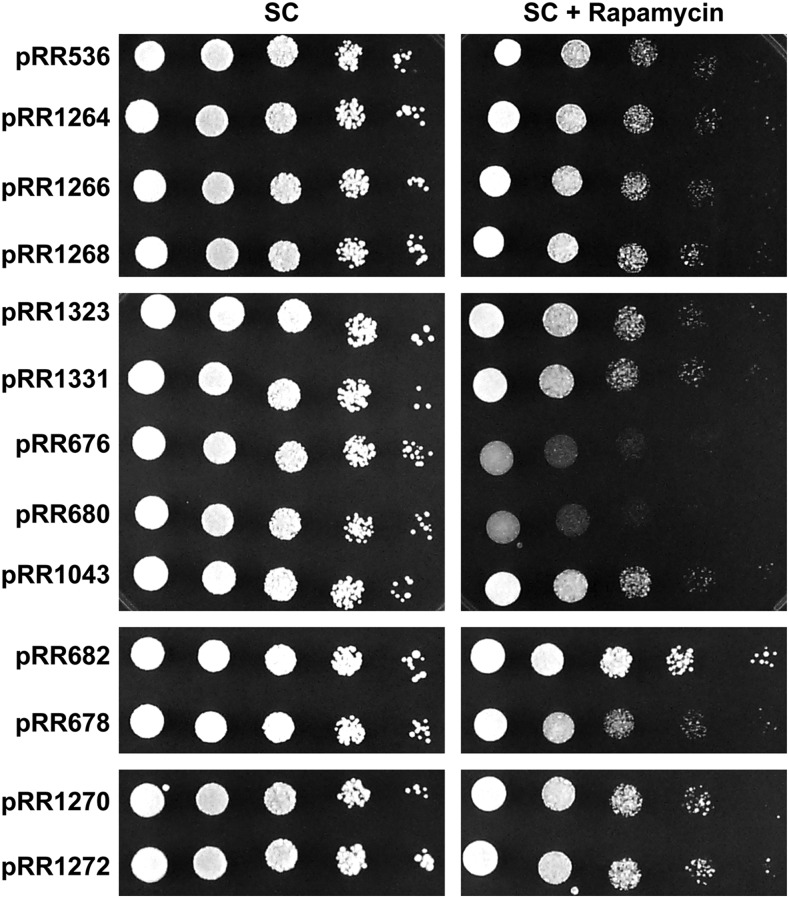
Rapamycin sensitivity of wild type and Gln3 amino acid substitution mutants. Transformants were spotted in succeeding 10-fold dilutions on synthetic complete medium, and the same medium containing 50 ng/ml rapamycin. The cells were cultured for just over 4 d at 30° C. The transformation recipient for these experiments was KHC2, a *gln3*Δ.

All of the mutant transformants, with two important exceptions, were no more rapamycin-sensitive than wild-type cells containing pRR536 (Figure 13). The first exception was with pRR680, containing the 13 serine/threonine to alanine substitutions in the Gln3_477–497_ region. The second, and more important, exception was with pRR676, in which the repeated RRSSRRSS was changed to RRAARRAA. Both constructs yielded highly rapamycin-sensitive transformants and, notably, they were equivalently rapamycin sensitive. The eight substitutions in pRR1043 adjacent to the RRSSRRSS sequence did not alter rapamycin sensitivity. Finally it is worth mentioning that none of the other substitution mutants, with the possible exception of pRR682, increased rapamycin resistance of transformants containing them as would be expected if they had adversely affected Gln3-mediated transcription.

### Alteration of the serine-rich region and the Gln3-mTor1 interaction site cumulatively abolishes cytoplasmic Gln3 localization

**Figure 14:**
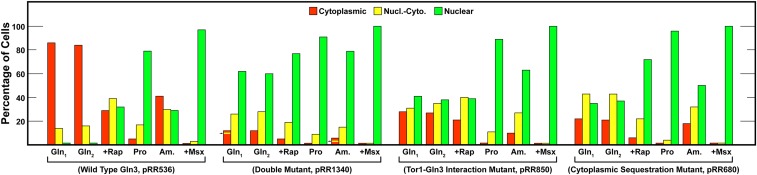
Cytoplasmic Gln3-Myc^13^ sequestration observed in transformants with individual alterations in the Gln3_479–493_ serine/threonine-rich region (pRR680), and the Gln3-mTor1 interaction site (pRR850, Rai *et al.* 2013) compared to that which occurs in a transformant containing both sets of alterations (pRR1340). The predicted level of cytoplasmic Gln3-Myc^13^ sequestration expected if the alterations are additively diminishing cytoplasmic Gln3-Myc^13^ sequestration are indicated with arrows and yellow lines in the glutamine and ammonia data derived with pRR1340 transformants. The experimental format and presentation of the data were as described in Figure 6.

During identification of the Gln3-mTor1 interaction site, we noticed that alteration of that site not only rendered Gln3 localization immune to the effects of rapamycin treatment, but also partially abolished its cytoplasmic sequestration in nitrogen-replete conditions. While this suggested that Gln3 localization was probably controlled by the cumulative participation of multiple independent pathways, there was no way of directly testing that hypothesis. Discovering that alteration of the Gln3_477-493_ serine-rich region also partially abolished cytoplasmic Gln3 sequestration without affecting its response to rapamycin afforded an opportunity of doing so. To this end, we constructed a double mutant consisting of the alterations contained in pRR680 and those in pRR850, which destroys the Gln3-mTor1 interaction site (Rai *et al.* 2013). As shown in Figure 14, the two single mutants exhibited remarkably similar phenotypes, except that Gln3-Myc^13^ localization responded to rapamycin treatment in cells containing pRR680, but not pRR850. Critically, cytoplasmic Gln3-Myc^13^ sequestration was only partially abolished in the single mutants provided with either glutamine or ammonia as sole nitrogen source. In sharp contrast, it was almost completely abolished in the double mutant transformant containing pRR1340 (Figure 14). Based on the degree of cytoplasmic Gln3-Myc^13^ localization in the single mutants relative to wild type, it is possible to predict the level of cytoplasmic Gln3-Myc^13^ in the double mutant if the mutated sites are acting additively. These levels are indicated with arrows and yellow lines in the responses of glutamine- or ammonia-grown cells containing pRR1340. The predicted and observed levels of cytoplasmic Gln3-Myc^13^ sequestration are nearly identical.

### Alteration of conserved proline-rich sequence affects both Gln3-Myc^13^ cytoplasmic sequestration and rapamycin response

**Figure 15:**
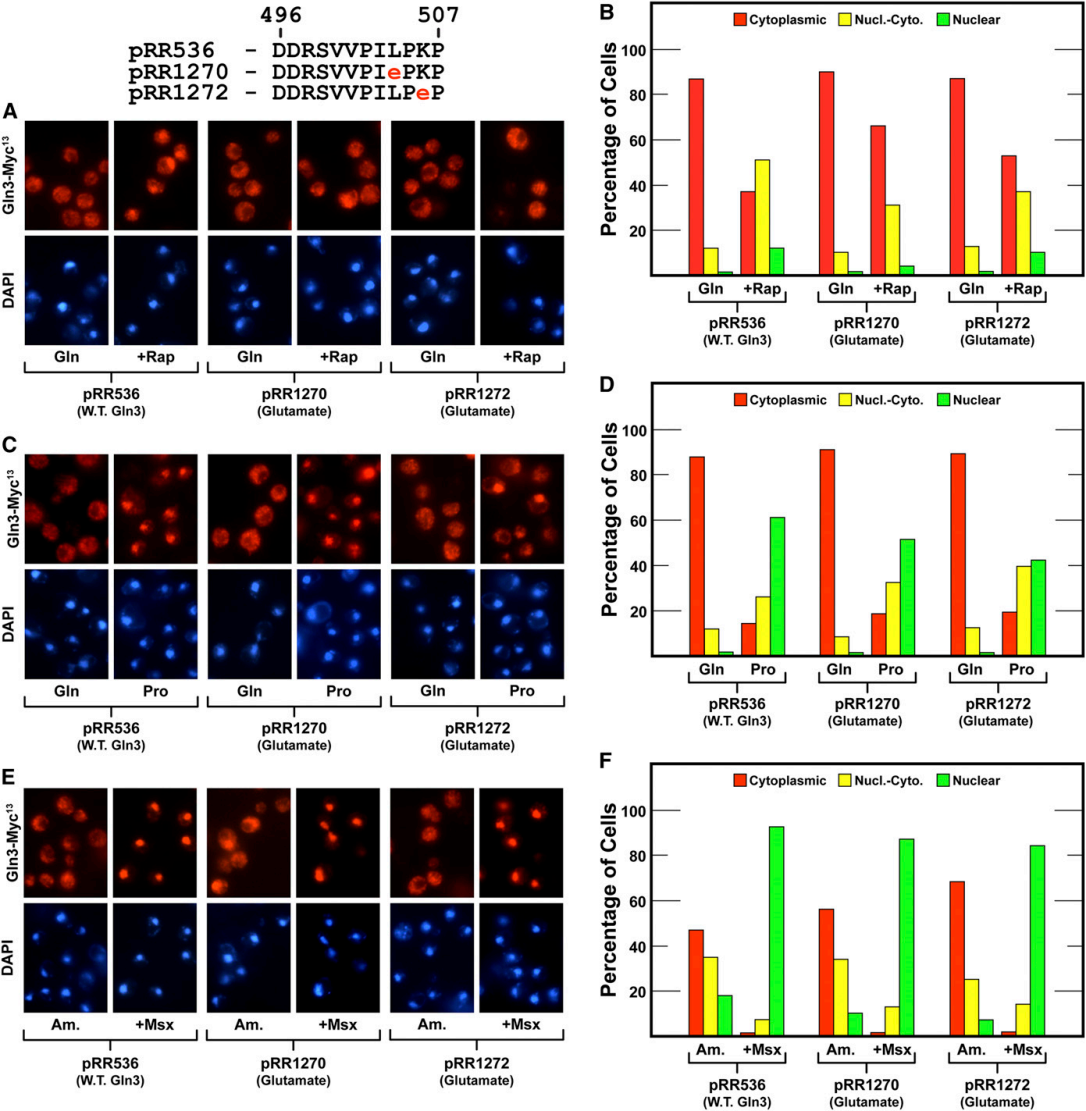
(A–F)Substitution of glutamate for the hydrophobic (pRR1270) and basic (pRR1272) residues in the proline-rich region between residues required for cytoplasmic Gln3-Myc^13^ sequestration and the Gln3 response to rapamycin treatment diminishes the rapamycin response. The experimental format and presentation of the data were as described in Figure 6.

At the C-terminus of the highly conserved serine- and basic amino acid-rich region was a nine residue sequence in which prolines alternated with other residues, *i.e.*, VPILPKPSP. To investigate this region, we substituted glutamate for the leucine or lysine residues in this region (Gln3_L504E_-Myc^13^, pRR1270; Gln3_K506E_-Myc^13^, pRR1272) (Figure 15). Both substitutions markedly decreased the ability of rapamycin to elicit nuclear Gln3-Myc^13^ localization (Figure 15, A and B). This phenotype contrasted with the wild-type responses of aspartate substitutions in the serine-rich region of Gln3 (Figure 6 and Figure 7 compared with Figure 15, C–F). Consistent with all of the earlier rapamycin-insensitive phenotypes, these alterations had no demonstrable effect on the ability of Msx to elicit high nuclear Gln3-Myc^13^ localization (Figure 15, E and F). Equally important, cytoplasmic Gln3-Myc^13^ sequestration was fully intact in both of these mutants. These results slightly extended the contiguous Gln3 domain required for a robust response to rapamycin.

## Discussion

**Figure 16:**
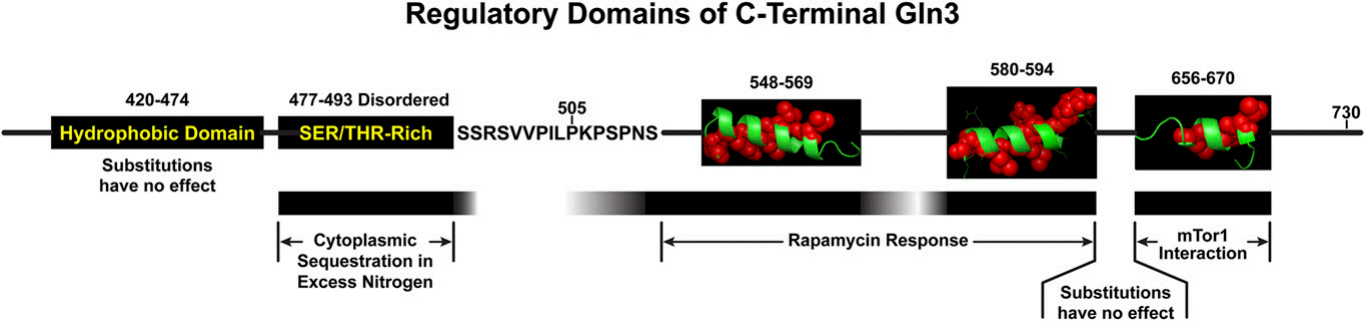
Schematic of the C-terminal regulatory region of Gln3. The black bars indicate strong requirements of the residues indicated at the top of the figure for the processes indicated. White regions in or between these bars indicate areas where substitutions had little to no effect. In instances where putative α-helices appear, the hydrophobic and nonhydrophobic residues appear in red balls and green ribbons, respectively (Rai *et al.* 2013, 2014).

Experiments presented in this work define a unique Gln3 17 amino acid peptide (Gln3_477–493_) that is required to sequester Gln3-Myc^13^ in the cytoplasm when cells are growing in nitrogen-rich medium (Figure 16). The site consists of a highly conserved serine/threonine-rich (12/15 residues) sequence flanked on either side by basic residues. Somewhat surprisingly, this region is not required for a Gln3 response to limiting nitrogen, rapamycin inhibition of mTorC1, or Msx inhibition of glutamine synthetase and ensuing glutamine starvation. Within this peptide is a repeated sequence: RRSSRRSS. When alanines are substituted for the four serines in this sequence (pRR676), a phenotype similar to that of substituting all 13 serine/threonine residues of Gln3_477-493_ (pRR680) was obtained. We speculate that the serine and threonine residues in Gln3_477–493_ are phosphorylated in cells provided with excess nitrogen, because substitution of a phosphorylation mimic, aspartate (pRR682 and pRR678), yielded the same level of cytoplasmic Gln3-Myc^13^ sequestration in nitrogen-rich medium as wild type, whereas alanine substitutions that cannot be phosphorylated yielded the mutant phenotype, *i.e.*, loss of cytoplasmic Gln3-Myc^13^ sequestration and rapamycin hypersensitivity (pRR680 and pRR676). Further, the slowest migrating Gln3-Myc^13^ species in western blots of the aspartate substituted mutants cited above was lost when alanine was substituted for these serines. The most slowly migrating Gln3-Myc^13^ species has been repeatedly shown to be the one that is most phosphorylated. The Gln3 site(s) and process(es) that control the phosphorylation of these residues remains a mystery.

Data presented above demonstrate that serines/threonines in Gln3_477–493_ must not only be capable of being phosphorylated, but also must act in concert with Gln3 sites phosphorylated by mTorC1 for Gln3-Myc^13^ to be effectively sequestered in the cytoplasm, as supported by the additive phenotypes of pRR850, pRR680, and pRR1340. However, the combined phosphorylation of these two targets is still insufficient to fully determine whether Gln3 enters the nucleus or is retained in the cytoplasm. Otherwise, the Gln3 localization in the single and double mutant transformants just cited should not have responded as they did to limiting nitrogen (proline grown), or Msx treatment. Further, also note that cytoplasmic Gln3-Myc^13^ sequestration is still not totally abolished even in the double mutant transformant growing in glutamine medium (Figure 14, pRR1340). In this regard it is pertinent to recall the important roles played by Ure2, and the influence of glutamine, on the course of events Gln3 follows once it is within the nucleus (Rai *et al.* 2015). What present and previously reported data do convincingly demonstrate is the presence of multiple distinctly regulated targets on the Gln3 molecule, which, by inductive reasoning, support the conclusion that nitrogen-response Gln3 localization and function are controlled by multiple, independent regulatory pathways.

One might conclude that Gln3_477–493_ contains the serines phosphorylated by mTor1, following its interaction with the Gln3_656–670_ site, because alteration of this site also results in a partial loss of cytoplasmic Gln3 sequestration (Rai *et al.* 2013). We do not favor this interpretation because: (i) abolishing the Gln3-mTor1 interaction site also abolished the ability of Gln3 to respond to rapamycin inhibition of mTorC1 (Rai *et al.* 2013), which did not occur in the Gln3_477–493_ mutants described in this work; and (ii) the electrophoretic mobility of Gln3-Myc^13^ increased when the mutant transformants were treated with rapamycin (Figure 11). By similar reasoning, if Gln3_477–493_ is phosphorylated, it is also unlikely to be the target of the rapamycin-elicited, Sit4-dependent Gln3 dephosphorylation and nuclear localization because a wild type rapamycin response occurred when aspartate was substituted for all of the serine residues in this site (Figure 8, Figure 9, and Figure 11). Further, Gln3-Myc^13^ only partially relocated to the nuclei of rapamycin-treated wild type cells. Yet when cytoplasmic Gln3-Myc^13^ retention was diminished by alanine substitutions in Gln3_477–493_, nuclear Gln3 localization correspondingly increased. This is the behavior expected when multiple regulatory pathways operate cumulatively to bring about an overall result.

Finally, this is the first time that Gln3 has been reported to potentially be a disordered molecule. It is fascinating that the three specific regulatory sites identified in Gln3 thus far occur in regions of the molecule that are predicted to be disordered. In the most extreme case, the Gln3_477–493_ residues required for cytoplasmic Gln3 sequestration are also those that exhibit the greatest probability of being disordered (see residues between the arrows in Figure 4C). Consistent with the predicted probability of this region being disordered are the observations that extensive substitutions between the regulatory sequences documented thus far exhibit wild type phenotypes (Gln3_421–473_ in this work, and in Rai *et al.* 2013). Yet the regulatory sites themselves are highly sensitive to amino acid substitutions. If the overall region was highly structured, these intersite substitutions would *a priori* be expected to exhibit something other than wild-type phenotypes. We speculate that the likelihood of the C-terminal region of Gln3 being disordered may be responsible for our ability to alter each of the individual regulatory sites in this region without telegraphing the effects of those alterations to the others. Only in the case of the short proline-rich region between the sites required for cytoplasmic Gln3 sequestration and rapamycin-responsiveness did we find substitutions that affected the functioning of two adjacent regulatory sites (Figure 15). We speculate that the core secondary structures of each of the sites that are the most sensitive to alteration (Figure 16) may initiate interactions with the regulatory molecules that target them followed by the acquisition of additional secondary structure emanating from the interactions themselves.
